# Association between postoperative hyponatremia and renal prognosis in major urologic surgery

**DOI:** 10.18632/oncotarget.20326

**Published:** 2017-08-18

**Authors:** Sehoon Park, Jung Nam An, Jung Pyo Lee, Yun Kyu Oh, Dong Ki Kim, Kwon Wook Joo, Yon Su Kim, Chun Soo Lim

**Affiliations:** ^1^ Department of Biomedical Sciences, Seoul National University College of Medicine, Seoul, Korea; ^2^ Department of Internal Medicine, Seoul National University Boramae Medical Center, Seoul, Korea; ^3^ Department of Internal Medicine, Seoul National University College of Medicine, Seoul, Korea; ^4^ Department of Internal Medicine, Seoul National University Hospital, Seoul, Korea

**Keywords:** hyponatremia, urology, acute kidney injury, end stage renal disease, surgery

## Abstract

Recent evidence for the clinical significance of postoperative hyponatremia after urologic surgeries remains scarce. We examined the incidence, risk factors, and outcomes of electrolyte imbalance in urologic surgery. Patients with newly developed hyponatremia, defined as a sodium level lower than 135 mEq/L within 7 days after surgery, were included in our study group. The primary outcome was progression to end-stage renal disease (ESRD). Secondary outcome was composition of progression to ESRD and creatinine doubling/eGFR halving from baseline. A survival analysis with a multivariable Cox proportional hazard model was performed. We included 9,206 cases of bladder, prostate, ureter, and kidney surgery. Incidence of new-onset postoperative hyponatremia was 15.4% (1,414/9,206). Postoperative hyponatremia mostly developed in patients with high-risk perioperative characteristics. The development of postoperative hyponatremia was independently associated with progression to ESRD (adjusted HR 1.343, 95% CI 1.082–1.680, *P =* 0.007). The secondary outcome was also related to the electrolyte imbalance in prostate (adjusted HR 1.729, 95% CI 1.145–2.612, *P =* 0.009) and kidney (adjusted HR 1.339, 95% CI 1.099–1.632, *P =* 0.004) surgery. Postoperative hyponatremia in urologic surgery was a common electrolyte imbalance in patients with high-risk perioperative status, and associated with worse renal prognosis.

## INTRODUCTION

Hyponatremia is the most common electrolyte imbalance in hospitalized patients [1, 2]. Previous studies have shown a close relationship between in-hospital hyponatremia and adverse clinical outcomes [2–6]. As surgery was found to be one of the main causes of in-hospital hyponatremia, some have investigated the clinical significance of postoperative hyponatremia in several fields, including neurosurgery, orthopedic operations, and urologic surgeries [3–5, 7–9]. However, recent studies considering electrolyte imbalance in urologic surgery have focused on the classic concept of transurethral-resection of prostate (TUR-P) syndrome [7, 8], although advances in technique have significantly decreased its incidence [10]. Hence, incidence and risk factors for postoperative hyponatremia in recent major urologic surgery need further investigation.

In addition, as the urinary system is directly manipulated and many disease entities are closely related to kidney function in urologic practice, few previous studies investigated AKI after urologic operation and demonstrated its significant impact on patient prognosis [11–13]. Considering that postoperative hyponatremia and AKI are both common in patients with perioperative risk factors [3, 5, 6, 9, 14, 15], one could assume that postoperative hyponatremia would also be related to worse postoperative prognosis [9]. However, whether postoperative hyponatremia is a risk factor for worse renal outcomes, independent from stages of kidney injury or other clinical factors, has not been determined.

In this study, we aimed to assess whether new-onset postoperative hyponatremia is associated with long-term renal prognosis in the urology field. We analyzed patients who underwent four major categories of recent urologic surgeries: bladder, prostate, ureter, and kidney, and investigated the prevalence, risk factors, and clinical outcomes of new-onset postoperative hyponatremia.

## RESULTS

### Study population and incidence of postoperative hyponatremia

**Figure 1:**
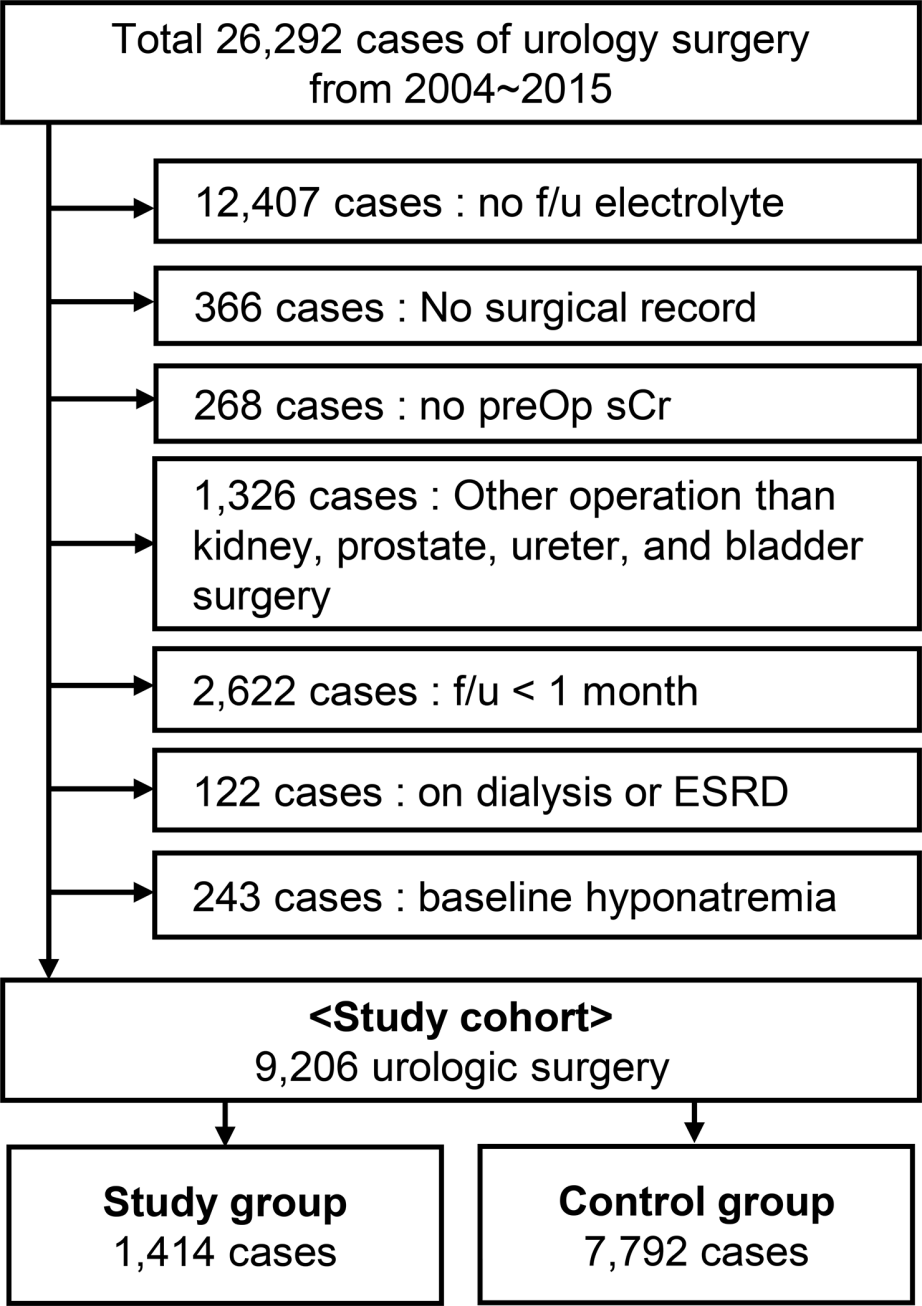
Flow diagram of the study population

**Figure 2:**
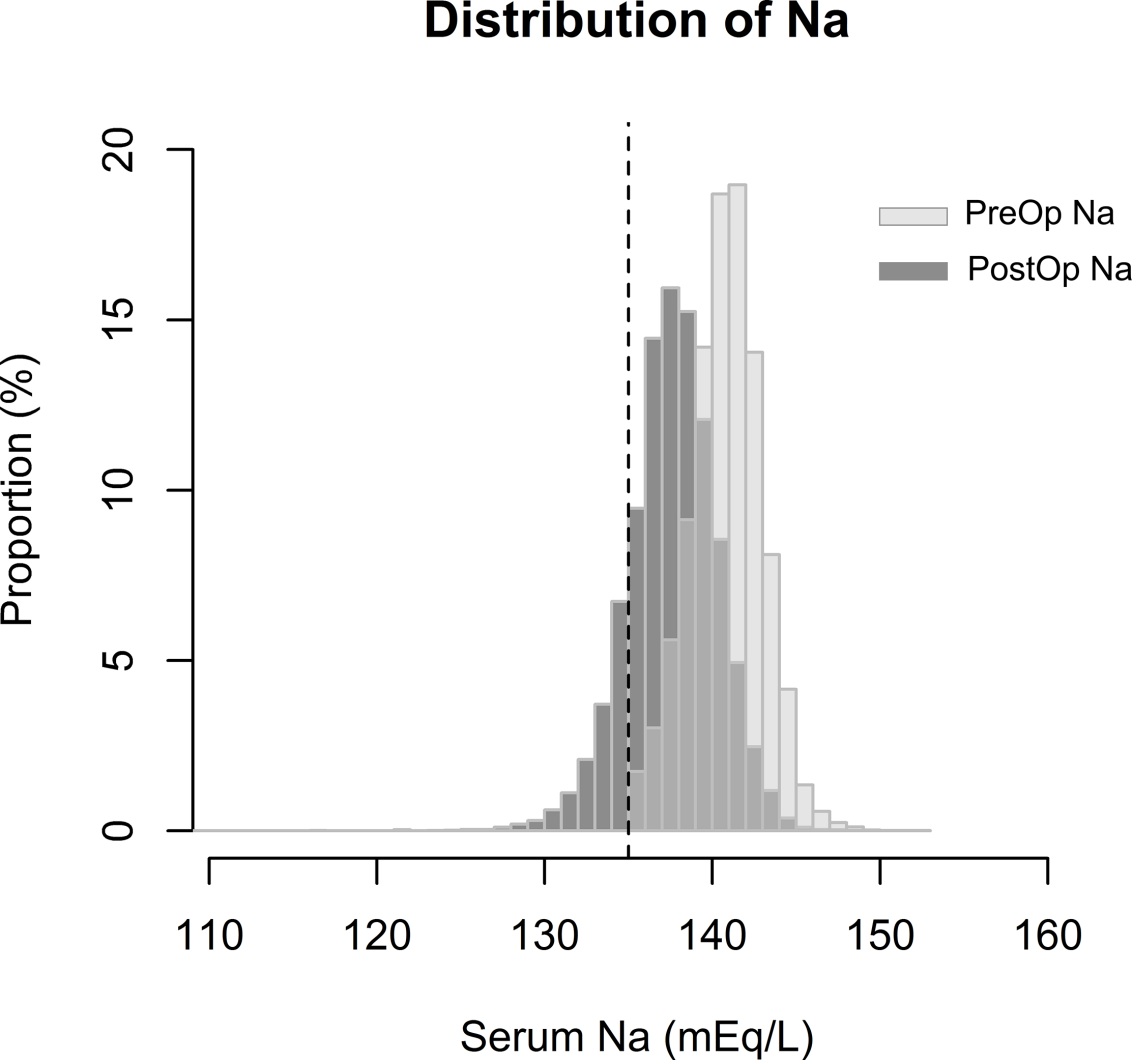
Distribution of lowest serum Na levels in the preoperative and postoperative periods in the study population The X-axis shows the serum Na level and Y-axis shows the proportion of patients identified with the serum Na level. Light grey bars indicate the distribution of preoperative Na (PreOp Na), and dark grey bars indicate the distribution of postoperative Na (PostOp Na) values of the study population. Dotted vertical line shows a serum Na level of 135 mEq/L, which was the criterion for hyponatremia in the study.

A total of 26,292 cases of urologic surgery were screened for study enrollment (Figure 1). Many patients were excluded due to non-available laboratory results of serum Na within 7 days after operation (*n* = 12,407), most of which were minor procedures such as vasectomy, circumcision, and wound repair. The two other main causes of exclusion were cases which could not be categorized into bladder, prostate, ureter, and kidney operations (*n* = 1,326) and those lost to follow up within 1-month post-surgery (*n* = 2,622). After exclusion, 9,206 cases of urologic surgery were included in our study. We identified major decrements in serum Na levels from baseline to the postoperative period in our study population (Figure 2). The incidence of new-onset postoperative hyponatremia was 15.4% (*n* = 1,414). Patients with hyponatremia development were included in the study group, while the others were considered as the control group. Most of the study group had mild to moderate hyponatremia, sodium range of 125–134 mEq/L (*n* = 1,312), and 102 patients had severe post-operative hyponatremia (Na < 125 mEq/mL).

### C*haracteristics of patients with postoperative hyponatremia*

Regarding preoperative patient characteristics (Table 1), the study group were more commonly female (*P* < 0.001), had a lower BMI (*P <* 0.001), and lower baseline hemoglobin (*P* < 0.001) and serum Na levels (*P* < 0.001) than the control group. Comorbidities, such as hypertension (*P* < 0.001), diabetes mellitus (*P =* 0.023), and cancer (*P* < 0.001), were more common in the study group. In contrast, baseline serum creatinine (*P* = 0.229) and eGFR (*P* = 0.164) were not significantly different between groups. No significant difference in preoperative medication use was identified between the study and the control groups.

**Table 1:** Baseline characteristics of study patients

	New-onset hyponatremia (+) (*N* = 1,414)	New-onset hyponatremia (-) (*N* = 7,792)	*P* value
Age (years)	65 (54–72)	65 (54–71)	0.065
Sex (Female)	375 (26.5)	1657 (21.3)	< 0.001
Body mass index (kg/m^2^)	24.0 (22.1–26.0)	24.3 (22.3–26.4)	< 0.001
Baseline laboratory results			
sCr (mg/dL)	0.92 (0.77–1.1)	0.92 (0.80–1.08)	0.229
eGFR (mL/min/1.73 m^2^)	68.7 (52.9–90.5)	67.6 (54.1–86.5)	0.164
Hemoglobin (g/dL)	13.2 (11.6–14.4)	13.6 (12.3–14.7)	< 0.001
Sodium (mEq/L)	140 (138–141)	141 (139–142)	< 0.001
Underlying comorbidities			
Cancer	1111 (78.6)	5691 (73.0)	< 0.001
Hypertension	551 (39.0)	2630 (33.8)	< 0.001
Diabetes mellitus	298 (21.1)	1441 (18.5)	0.023
Chronic kidney disease	502 (35.5)	2730 (35.0)	0.735
Liver cirrhosis	67 (4.7)	312 (4.0)	0.201
Heart failure	104 (7.4)	491 (6.3)	0.138
Preoperative use of			
ACE I/ARBs	165 (11.7)	853 (10.9)	0.426
Diuretics	150 (10.6)	723 (9.3)	0.116
SSRI/SNRIs	14 (1.0)	97 (1.2)	0.419
NSAIDs	273 (19.3)	1649 (21.2)	0.114
Antipsychotics	11 (0.8)	43 (0.6)	0.306
Benzodiazepines	42 (3.0)	242 (3.1)	0.786
Antiepileptic agents	46 (3.3)	211 (2.7)	0.252
Proton pump inhibitor	95 (6.7)	578 (7.4)	0.353
Thyroidal hormones	19 (1.3)	92 (1.2)	0.605
Anti–thyroidal medications	1 (0.1)	13 (0.2)	0.393
Oral steroids	46 (3.3)	282 (3.6)	0.495

sCr, serum creatinine, eGFR, estimated glomerular filtration rate, ACE I, angiotensin converting enzyme inhibitor, ARB, angiotensin receptor blocker, SSRI, selective-serotonin reuptake inhibitor, SNRI, serotonin norepinephrine reuptake inhibitor, NSAID, non-steroidal anti-inflammatory drug

Values were presented as *n* (%) for categorical variables, and median (25% to 75% interquartile) for continuous variables (all data in the table showed non-normal distribution).

With regard to operation type (Table 2), the incidence of postoperative hyponatremia was significantly different according to the surgery category. Among the four operation categories, hyponatremia was most frequently (27%) observed in patients of kidney operation, while incidences of the electrolyte imbalance were similar in bladder (12.5%), prostate (12.3%), and ureter (10.9%) operations.

**Table 2:** Categories of operations and incidence of hyponatremia

Operation category	^a^Incidence of hyponatremia	New-onset hyponatremia (+) (*N* = 1,414)	New-onset hyponatremia (-) (*N* = 7,792)	*P* values
Bladder	11.1%	201 (14.2)	1,613 (20.7)	< 0.001
TUR-B	8.1%	86 (6.1)	980 (12.6)	
Cystolithotripsy	4.8%	3 (0.2)	60 (0.8)	
Cystectomy	18.9%	101 (7.1)	433 (5.6)	
Cystocele repair	1.8%	1 (0.1)	60 (0.8)	
Transurethral coagulation	20%	3 (0.2)	12 (0.2)	
Cystostomy	0%	0 (0.0)	4 (0.1)	
Other cystoscopy	6.1%	1 (0.1)	13 (0.2)	
miscellaneous	10.5%	6 (0.4)	51 (0.7)	
Prostate	10.9%	323 (22.8)	2,634 (33.8)	< 0.001
TUR-P, HoLEP etc.	8.8%	52 (3.7)	540 (6.9)	
Radical prostatectomy	11.5%	271 (19.2)	2,084 (26.7)	
miscellaneous	0%	0 (0.0)	10 (0.1)	
Ureter	9.8%	59 (4.2)	540 (6.9)	< 0.001
Stone removal	9.1%	30 (2.1)	301 (3.9)	
Ureterectomy	22.2%	4 (0.3)	14 (0.2)	
Ureterotomy	7.8%	4 (0.3)	47 (0.6)	
Ureteral stent insertion/removal	13%	13 (0.9)	87 (1.1)	
Other ureteroscopy	5.4%	3 (0.2)	56 (0.7)	
miscellaneous	12.5%	5 (0.4)	35 (0.4)	
Kidney	21.7%	831 (58.8)	3,005 (38.8)	< 0.001
Partial nephrectomy	20.0%	278 (19.7)	1,110 (14.2)	
Unilateral radical nephrectomy	24.9%	525 (37.1)	1,583 (20.3)	
Bilateral nephrectomy	0%	0 (0.0)	1 (0.0)	
Nephrolithotomy	8.8%	27 (1.9)	280 (3.6)	
Pyeloplasty	3.1%	1 (0.1)	31 (0.4)	

TUR-B, transurethral bladder resection, TUR-P, transurethral prostate resection, HoLEP, Holmium Laser Enucleation of the prostate

Included numbers of surgery cases were presented as *n* (%).

^a^Incidence of postoperative hyponatremia in each subtypes of surgery (in each row) was shown.

In addition, perioperative characteristics (Table 3) revealed that new-onset postoperative hyponatremia was associated with longer surgical/anesthesia time (*P* < 0.001) and higher preoperative risk stratification scores in terms of ASA classification (*P* < 0.002). The study group more frequently received common volume expanders including normal saline (*P* < 0.001), Hartman solution (*P* = 0.010), and hydroxyethyl starch (*P* < 0.001), and were infused with larger volumes of Hartman solution when used (*P <* 0.001). Increased RBC transfusion amounts were also observed in patients with postoperative hyponatremia (*P* < 0.001).

**Table 3:** Perioperative findings of study patients

	New-onset hyponatremia (+) (*N* = 1,414)	New-onset hyponatremia (-) (*N* = 7,792)	*P* value
Surgery type			0.906
Open surgery	713 (50.4)	3936 (50.5)	
Laparoscopic surgery	183 (12.9)	1006 (12.9)	
Endoscopic surgery	335 (23.7)	1891 (24.3)	
Robot-assisted surgery	183 (12.9)	959 (12.3)	
Anesthesia type			0.067
Spinal	117 (8.4)	819 (10.6)	
General	1,267 (91.0)	6,791 (88.3)	
Others (MAC, epidural, etc.)	8 (0.6)	80 (1.1)	
ASA classification			0.002
0	4 (0.3)	29 (0.4)	
1	455 (33.3)	2,629 (34.7)	
2	793 (58.0)	4,470 (59.1)	
3	112 (8.2)	434 (5.7)	
≥ 4	3 (0.2)	4 (0.1)	
NYHA classification			0.092
1	1,182 (89.5)	6,683 (91.2)	
2	117 (8.9)	574 (7.8)	
≥ 3	22 (1.7)	73 (1.0)	
Anesthesia time (minutes)	190 (145–250)	175 (115–235)	< 0.001
< 120	194 (14.0)	1994 (26.0)	
≥ 120, and < 180	381 (27.4)	1894 (24.7)	
≥ 180	813 (58.6)	3777 (49.3)	
In non-general anesthesia (minutes)	145 (85–170)	115 (60–160)	< 0.001
In general anesthesia (minutes)	200 (150–255)	185 (125–245)	< 0.001
Surgical time (minutes)	147 (109–200)	135 (77–190)	< 0.001
< 120	438 (31.6)	3176 (41.5)	
≥ 120, and < 180	466 (33.6)	2264 (29.5)	
≥ 180	483 (34.8)	2222 (29.0)	
In non-general anesthesia (minutes)	109 (50–135)	85 (35–125)	0.001
In general anesthesia (minutes)	155 (110–209)	140 (87–197)	< 0.001
Emergency operation	3 (0.2)	25 (0.3)	0.505
Intraoperative use of intravenous fluid			
Balanced salt solution			
Number of cases	374 (26.4)	2,065 (26.5)	0.968
Amount of fluid use (mL)	800 (500–1,200)	800 (500–1,150)	0.863
Hartman solution			
Number of cases	1,175 (83.1)	6,244 (80.1)	0.010
Amount of fluid use (mL)	850 (600–1,300)	800 (500–1,200)	< 0.001
Normal saline			
Number of cases	531 (37.6)	2,357 (30.2)	< 0.001
Amount of fluid use (mL)	700 (400–1,000)	700 (400–1,000)	0.136
Hydroxyethyl starch			
Number of cases	469 (33.2)	2,147 (27.6)	< 0.001
Amount of fluid use (mL)	500 (500–825)	500 (500–1,000)	0.394
Intraoperative RBC transfusion			< 0.001
1–2 pack	114 (8.1)	347 (4.5)	
3–4 pack	38 (2.7)	125 (1.6)	
5–9 pack	9 (0.6)	43 (0.6)	
> 9 pack	3 (0.2)	9 (0.1)	
Inotropic agents use at Op day	18 (1.3)	118 (1.5)	0.489

MAC, monitored anesthesia care, RBC, red blood cell, Op, operation

Values were presented as *n* (%) for categorical variables, and median (25% to 75% interquartile) for continuous variables (all data in the table showed non-normal distribution).

Lastly, postoperative characteristics were also significantly different between the study and control groups (Table 4). Postoperative hyponatremia was significantly related to acute kidney injury (*P* < 0.001). The study group also had a longer hospital stay (*P* < 0.001), but the frequency of postoperative ICU admission was not significantly different between groups (*P* = 0.114).

**Table 4:** Postoperative findings of study patients

	New-onset hyponatremia (+) (*N* =1,414)	New-onset hyponatremia (-) (*N* = 7,792)	*P* value
Acute kidney injury			< 0.001
Stage 1	415 (30.3)	1,410 (20.9)	
Stage 2	26 (1.9)	78 (1.2)	
Stage 3	49 (3.6)	151 (2.2)	
sCr (mg/dL)	1.10 (0.91–1.40)	1.03 (0.90–1.28)	< 0.001
eGFR (mL/min/1.73 m^2^)	55.0 (40.0–74.6)	59.0 (45.5–74.9)	< 0.001
Sodium (mEq/L)	133 (132–134)	137 (136–139)	< 0.001
Hemoglobin (g/dL)	10.9 (9.6–12.0)	11.4 (10.2–12.6)	< 0.001
Sodium decrement (mEq/L)	7.0 (9.0–5.0)	3.0 (5.0–2.0)	< 0.001
Hemoglobin decrement (g/dL)	2.2 (1.1–3.3)	2.1 (1.1–3.2)	0.038
Body weight change (kg)	0.0 (–1.0 – 1.0)	0.0 (–1.0 – 1.0)	0.511
Days of hospital stay (days)	6 (5–9)	5 (4–8)	< 0.001
Postoperative ICU admission	14 (1.0)	48 (0.6)	0.114

sCr, serum creatinine, eGFR, estimated glomerular filtration rate, ICU, intensive care unit.

Values were presented as *n* (%) for categorical variables, and median (25% to 75% interquartile) for continuous variables (all data in the table showed non-normal distribution).

### Risk factors for postoperative hyponatremia

Outcomes related to risk factors for new-onset postoperative hyponatremia are summarized in Table 5. Multivariable analysis revealed age (adjusted OR 1.013, 95% CI 1.007–1.018, *P* < 0.001), female sex (adjusted OR 1.216, 95% CI 1.045–1.525, *P* = 0.011), history of diabetes mellitus (adjusted OR 1.317, 95% CI 1.116–1.554, *P* = 0.001), and cancer (adjusted OR 1.248, 95% CI 1.067–1.459, *P* = 0.006) were independent risk factors for hyponatremia development. Regarding laboratory results, baseline serum Na (adjusted OR 0.829, 95% CI 0.803–0.856, *P* < 0.001), and anemia at baseline (adjusted OR 1.311, 95% CI 1.139–1.509, *P* < 0.001) were significantly associated with new-onset postoperative hyponatremia. In addition, kidney operation (adjusted OR 2.765, 95% CI 2.388–3.202, *P* < 0.001), longer surgical time (adjusted OR 1.109, 95% CI 1.063–1.157, *P* < 0.001), ASA class III or higher (adjusted OR 1.621, 95% CI 1.372–1.915, *P* < 0.001), and intraoperative use of normal saline (adjusted OR 1.236, 95% CI 1.079–1.416, *P* = 0.002) or Hartman solution (adjusted OR 1.319, 95% CI 1.107–1.573, *P* = 0.002) were independently related to the presence of postoperative hyponatremia. Furthermore, postoperative AKI was strongly associated with the development of electrolyte imbalance and the risk showed increasing tendency in higher AKI stages.

**Table 5:** Risk factors for new-onset postoperative hyponatremia

Variables	^b^Adjusted OR (95% CI)	*P* value
Age (1-year increment)	1.013 (1.007–1.018)	< 0.001
Female sex (vs. male)	1.216 (1.045–1.414)	0.011
Body mass index (1 kg/m^2^ increment)	0.999 (0.999–1.000)	< 0.001
Cancer (vs. no)	1.248 (1.067–1.459)	0.006
Hypertension (vs. no)	1.139 (0.995–1.305)	0.060
Diabetes mellitus (vs. no)	1.317 (1.116–1.554)	0.001
Baseline Na (1 mEq/L increment)	0.829 (0.803–0.856)	< 0.001
Baseline anemia (hemoglobin < 12 g/dL)	1.311 (1.139–1.509)	< 0.001
Kidney operation (vs. other surgery)	2.765 (2.388–3.202)	< 0.001
Surgery time (1-hour increment)	1.109 (1.063–1.157)	< 0.001
NYHA classification (≥ 2)	1.159 (0.905–1.485)	0.243
ASA classification (≥ class III)	1.621 (1.372–1.915)	< 0.001
Use of Hartman solution (vs. no)	1.319 (1.107–1.573)	0.002
Use of hydroxyethyl starch (vs. no)	1.139 (0.978–1.325)	0.094
Use of normal saline (vs. no)	1.236 (1.079–1.416)	0.002
Transfusion amount of RBC (1-pack increment)	1.021 (0.984–1.060)	0.274
Acute kidney injury (vs. no acute kidney injury)		
Stage 1	1.433 (1.232–1.666)	< 0.001
Stage 2	1.649 (1.023–2.657)	0.040
Stage 3	1.724 (1.214–2.447)	0.002

OR, odds ratio, CI, confidence interval

^b^Adjusted simultaneously with all variables in the table and multivariable logistic regression test was performed.

### Risk of progression to ESRD in patients with postoperative hyponatremia

**Figure 3:**
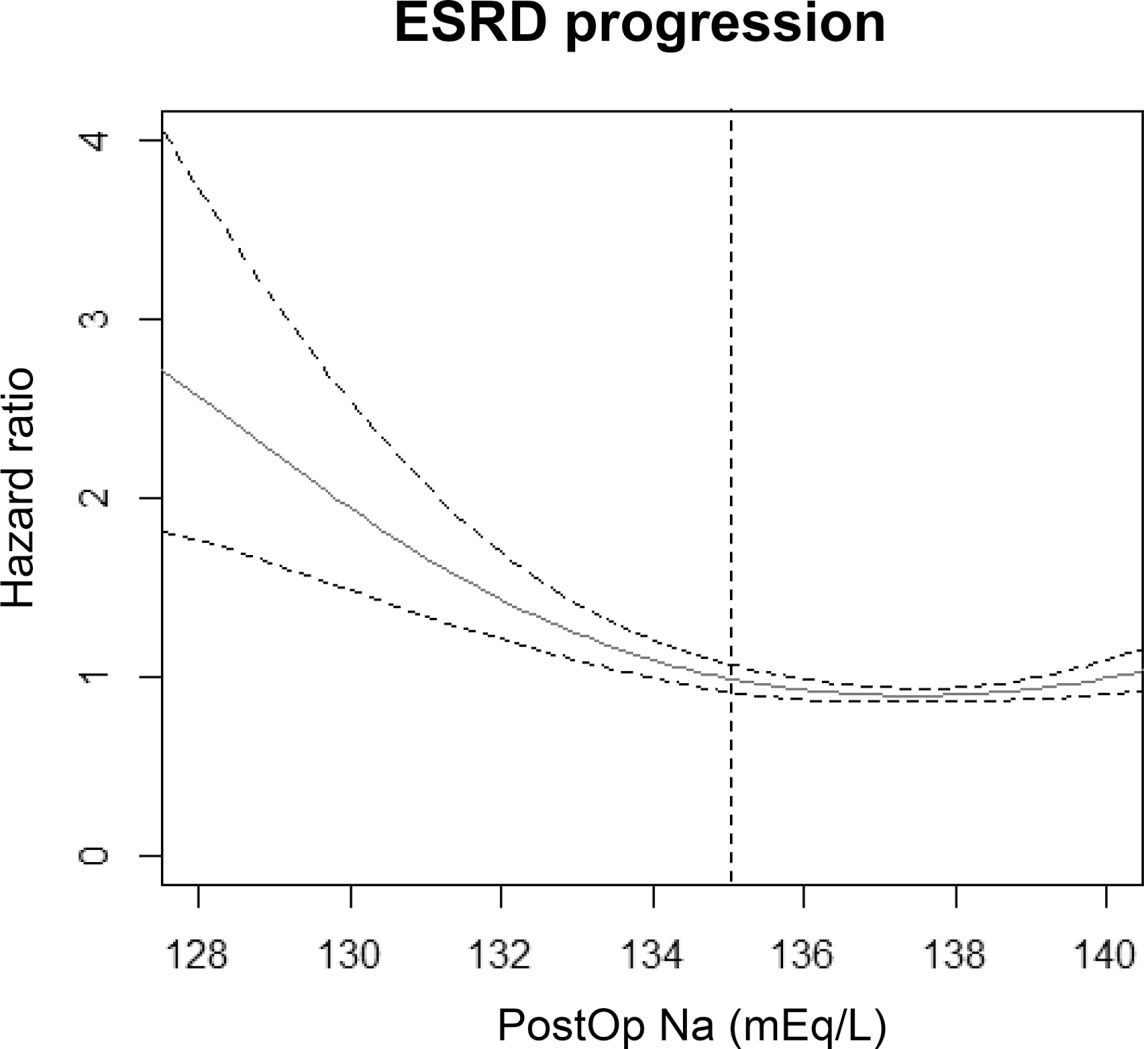
Cox proportional hazard models with panelized smoothing splines showing the association of postoperative Na level and risk of progression to ESRD The hazard ratio was calculated using the Na level with lowest risk for ESRD as the reference value. The X-axis shows the lowest postoperative sodium level within 7 days from surgery and the Y-axis shows the hazard ratios. Broken lines signify 95% confidence interval. The vertical broken line is drawn at a sodium level of 135 mEq/L, which was the criterion used to define postoperative hyponatremia in the study.

Six-hundred and four cases of progression to ESRD were documented, 122 (8.6%) in the study group, and 482 (6.2%) in the control group. The median duration to progression to ESRD was 0.5 (0.2–2.0) years in the study group, which was shorter than the median duration in the control group (1.1 [0.3–3.0] years). When we plotted the risk of ESRD regarding postoperative Na levels, an increased risk of progression to ESRD was observed with lower levels of postoperative serum Na (Figure 3). Moreover, after adjustment with multiple risk factors, which were revealed to be significant in the above analyses, development of postoperative hyponatremia was an independent risk factor associated with progression to ESRD (adjusted HR 1.343, 95% CI 1.082–1.680, *P* = 0.007).

### Long-term renal outcomes of postoperative hyponatremia

**Figure 4:**
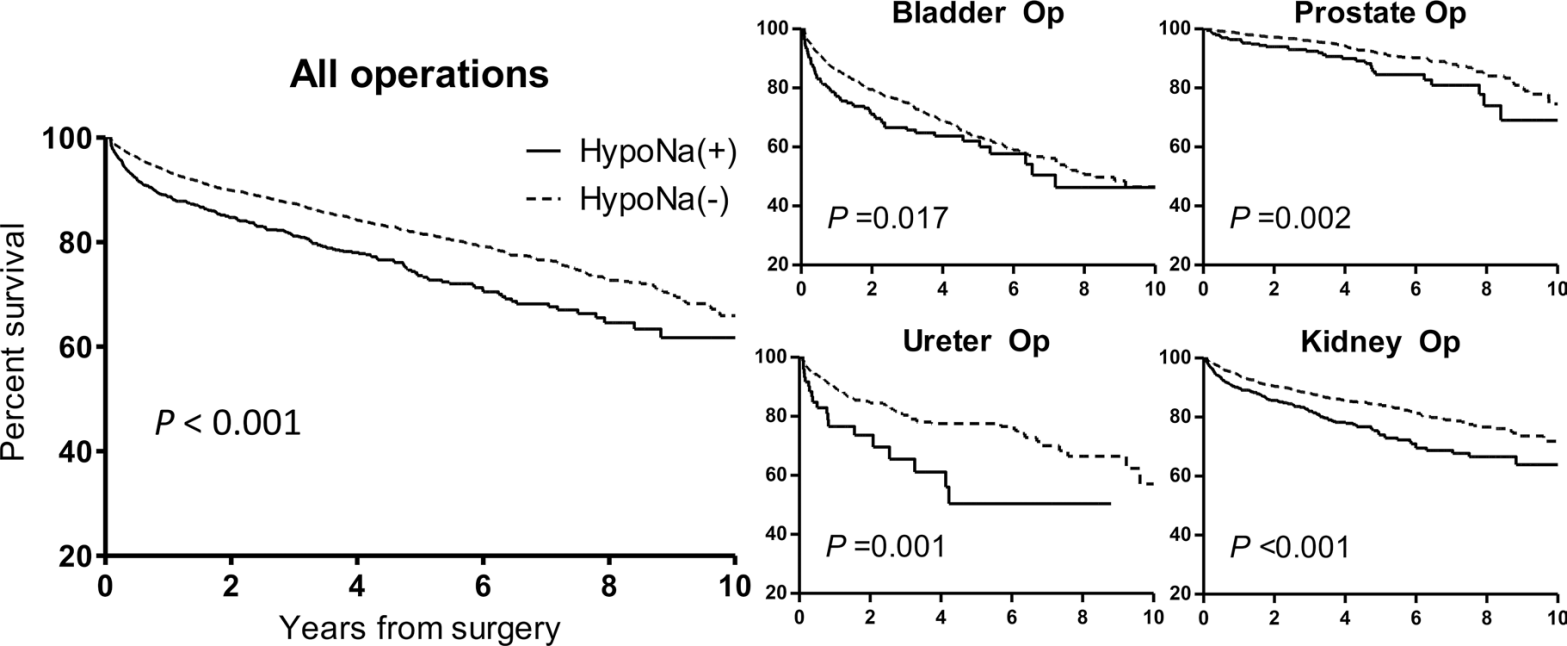
Kaplan-Meier survival curves of the composite renal outcome in all operations and each category of surgery, according to the presence of new-onset postoperative hyponatremia The composite renal outcome consisted of events of progression to ESRD, serum creatinine doubling or estimated glomerular filtration rate halving form baseline. The X-axis shows the duration from the surgery by years, and the Y-axis shows the percent survival of the composite renal outcome. HypoNa (+) indicates the study group with postoperative hyponatremia, and HypoNa (-) indicates the control group without hyponatremia. The solid line represents the survival curve of the study group and the fine dotted line shows the survival curve of the control group.

During a median follow-up duration of 2.6 (1.0–4.9) years in the study cohort, there were 1,366 secondary composite outcomes, which was the long-term renal prognosis outcome consisting of the composition of progression to ESRD and doubling of sCr or halving of eGFR from baseline. Among them, patients with postoperative hyponatremia showed worse secondary outcomes than the others (Figure 4). Our multivariable analysis revealed that development of postoperative hyponatremia remained a significant risk factor (adjusted HR 1.331, 95% CI 1.148–1.542, *P* < 0.001) for worse renal prognosis along with other well-known risk factors, such as old age, male sex, preoperative anemia, preoperative or postoperative kidney function measured by serum creatinine level, and history of diabetes mellitus (Table 6).

**Table 6:** Clinical characteristics related to long-term renal prognosis in the study cohort

Variables	^a^Adjusted HR	95% CI	*P* value
Age (years)	1.033	1.027–1.039	< 0.001
Female sex	0.700	0.593–0.827	< 0.001
Hypertension	1.025	0.897–1.172	0.713
Diabetes mellitus	1.427	1.216–1.675	< 0.001
Preoperative severe anemia (Hb < 8.0 g/dL)	1.282	1.036–1.586	0.022
Preoperative creatinine (mg/dL)	2.022	1.711–2.389	< 0.001
Postoperative creatinine (mg/dL)	1.010	1.004–1.017	0.003
Intraoperative use of normal saline	1.030	0.905–1.172	0.656
Postoperative hyponatremia	1.331	1.148–1.542	< 0.001

HR, hazard ratio, CI, confidence interval, hb, hemoglobin

^a^Adjusted with organ of surgery (bladder, prostate, ureter, kidney), surgery type (open, laparoscopic, endoscopic, and robot-assisted surgery), age, sex, history of hypertension, diabetes mellitus, preoperative severe anemia, intraoperative use of normal saline, preoperative and postoperative serum creatinine level.

Considering the heterogeneity of our study population, we further divided our study cohort according to clinically important characteristics and assessed the effect of new-onset postoperative hyponatremia in each subpopulation (Table 7). The association between postoperative hyponatremia and worse renal outcome was greater in younger, male patients. The association was significant regardless of preexisting reduced kidney function (eGFR < 60 mL/min/1.73 m^2^). Postoperative hyponatremia for prostate (adjusted HR 1.729, 95% CI 1.145–2.612, *P* = 0.009) and kidney (adjusted HR 1.339, 95% CI 1.099–1.632, *P* = 0.004) surgery patients was significantly associated with a worse secondary outcome.

**Table 7:** Association of postoperative hyponatremia and long-term renal prognosis in various subgroups

Subpopulation	^a^Adjusted HR	95% CI	*P* value
Age ≥ 70 (*N* = 3, 060)	1.088	0.861–1.376	0.479
Age 50–70 (*N* = 4, 670)	1.490	1.205–1.843	< 0.001
Age < 50 (*N* = 1, 476)	1.918	1.245–2.957	0.003
Female (*N* = 2, 032)	1.227	0.873–1.725	0.239
Male (*N* = 7, 174)	1.362	1.156–1.605	< 0.001
eGFR < 60 (*N* = 3, 232)	1.299	1.066–1.584	0.010
eGFR ≥ 60 (*N* = 5, 974)	1.425	1.142–1.778	0.002
Bladder operation (*N* = 1,814)	1.118	0.830–1.508	0.463
Prostate operation (*N* = 2,957)	1.729	1.145–2.612	0.009
Ureter operation (*N* = 599)	1.634	0.899–2.971	0.107
Kidney operation (*N* = 3,836)	1.339	1.099–1.632	0.004

HR, hazard ratio, CI, confidence interval, eGFR, estimated glomerular filtration rate (mL/min/1.73 m^2^)

^a^Adjusted with organ of surgery (bladder, prostate, ureter, kidney), surgery type (open, laparoscopic, endoscopic, and robot-assisted surgery), age, sex, history of hypertension, diabetes mellitus, preoperative severe anemia, intraoperative use of normal saline, preoperative and postoperative serum creatinine level.

## DISCUSSION

In this study, we examined the incidence, risk factors, and outcomes of new-onset postoperative hyponatremia in urologic surgery. Hyponatremia was a common electrolyte disorder after major urologic operations, especially in patients with high-risk perioperative characteristics. Decreased sodium level was associated with increased risk of progression to ESRD. A worse composite renal outcome was related to the development of postoperative hyponatremia, and the results remained significant even after adjustment for multiple clinical factors.

Previous studies showed that postoperative hyponatremia was largely a phenomenon caused by surgical stress, and that pathophysiologic changes in antidiuretic hormonal release play an important role in water retention and decreased serum Na [16]. In a similar vein, patients with comorbidities and other risk factors were considered to be vulnerable to such stress during operation, and, therefore, to the development of postoperative hyponatremia. Our study results demonstrate that high-risk perioperative status was closely related to the development of postoperative hyponatremia, agreeing with previous studies. Predominant use of normal saline, Hartman solution, and hydroxyethyl starch in the study group might also have been related to hemodynamic instability during surgery, as these fluids are commonly used for intraoperative volume expansion. Longer surgical time was also significantly associated with the development of electrolyte disturbance, as the patients might have experienced more hemodynamic changes during the perioperative period. Additionally, women are known to be prone to develop postoperative hyponatremia due to differences in hormone response and smaller total distribution volume; our findings correspond with this knowledge [5, 6]. Moreover, many known risk factors, such as older age and chronic comorbidities, were also confirmed to be independently associated with new-onset postoperative hyponatremia in urologic surgery [3, 5, 6, 9].

Furthermore, we demonstrated that postoperative hyponatremia was closely related to poor prognosis in regards to progression to ESRD, and composition of adverse renal outcomes. Considering that the electrolyte imbalance was an independent predictor for worse prognosis, even after adjustment for multiple risk factors, clinicians should closely monitor possible kidney function deterioration in patients with postoperative hyponatremia. In addition, pre- and post-operative sCr levels or AKI might have been the most commonly considered predictor for renal outcome, but the association of postoperative hyponatremia with poor renal prognosis remained significant, even after adjusting for sCr values. The reasons for the above results include: 1) as mentioned above, postoperative hyponatremia is related to stressful conditions during surgery [16], implying that patients with an electrolyte imbalance would suffer from more postoperative complications and have a worse clinical prognosis; 2) as patients with postoperative hyponatremia had more comorbidities, the electrolyte imbalance might be an indicator of severe illness; however, our multivariable analysis suggested that new-onset postoperative hyponatremia was an independent risk factor, even from pre- and post-operative creatinine levels, for worse renal outcome; 3) patients with postoperative hyponatremia had longer hospital stays, so exposure to additional in-hospital complications such as infection could have been attributed to adverse outcomes [3, 4, 9]; and 4) although rare, direct complications of hyponatremia, such as neurologic symptoms, might also have contributed to a worse prognosis [17, 18].

The association between the composite renal prognosis and new-onset postoperative hyponatremia differed among subpopulations in our study. Although young, male patients were less prone to experience postoperative hyponatremia, when developed, the clinical significance of the electrolyte imbalance was greater in these patients. Because patients with postoperative hyponatremia who underwent prostate or kidney operation had a worse renal prognosis, clinicians should pay careful attention to the electrolyte imbalance in these patients. Particularly, as preserving kidney function is a critical problem for patients who undergo nephrectomy, which was the most common kidney surgery in this study cohort, not only the pre- and post-operative serum creatinine levels, but also the electrolyte imbalance after surgery should be considered as predictive of poor renal prognosis. Moreover, although technical advances have decreased the incidence of TUR-P syndrome [10], postoperative hyponatremia remains an important risk factor for worse renal outcome in prostate operations. The types of bladder and ureter operations included in our study were relatively minor surgeries compared to the others, and renal deterioration occurred less in these patients, which may be why the association between hyponatremia development and worse composite renal outcome was not prominent in these patients.

Several limitations should be noted in our study. First, as this was a retrospective, observational study, our study cohort did not receive standardized treatment or follow up after their operations. In addition, detailed surgical category and postoperative fluid administration strategies were heterogeneous. However, the study design enabled us to evaluate the clinical significance of postoperative hyponatremia in a large cohort of urologic surgery cases with sufficient statistical power. Second, minor surgeries without follow up samples of serum electrolytes were excluded as were some operation categories. Hence, our findings may not be applicable to these other surgery categories. Third, the exact mechanism for worsened prognosis after postoperative hyponatremia could not be determined by our study results. Lastly, as this study was a bi-center study in a single nation, the study results may not be generalizable to patients of other centers with a different medical environment.

In conclusion, postoperative hyponatremia in urologic surgery was common and was related to high-risk perioperative characteristics. Considering that the presence of postoperative hyponatremia was strongly associated with consequent worse renal prognosis, clinicians should carefully consider such adverse outcomes in patients with this electrolyte imbalance, and evaluate the cause of hyponatremia and coexisting comorbidities in these patients.

## MATERIALS AND METHODS

### Ethics statement

This study was approved by the institutional review boards of Seoul National University Hospital (J-1407-145-597) and Seoul National University Boramae Medical Center (16-2014-96). This study was conducted following the principles of the Declaration of Helsinki. As the study was a retrospective, observational study without medical intervention, informed consent was waived.

### Study cohort

The study was a retrospective cohort study including patients from two tertiary hospitals in Korea. The inclusion criteria were bladder, prostate, ureter, and kidney surgery cases performed in the urologic department, with patients aged ≥ 18 years and available postoperative follow-up serum Na level within 7 days. The following patients were excluded from the study: 1) those with preexisting ESRD; 2) with no preoperative sCr within 1 month; 3) with no documented surgical record; 4) other urology surgeries than the four major categories identified in this study or complex operation cases involving multiple organs, 5) those lost to follow up less than 1 month post-operation due to causes other than death or the start of RRT, and 6) patients who had baseline hyponatremia. The study group consisted of patients with newly developed postoperative hyponatremia, defined as at least one serum sodium level lower than 135 mEq/L within 7 days after surgery, and the others were included in the control group.

### Data collection

We collected the following preoperative demographic, laboratory, and clinical information of study subjects by EHR review: age; sex; weight; height; body mass index; baseline and follow-up levels of serum sodium and potassium; all measured values of sCr in each patient and eGFR values, calculated using the Chronic Kidney Disease Epidemiology Collaboration (CKD-EPI) equation [19], and baseline hemoglobin levels. Comorbidity, including cancer, hypertension, and diabetes, were collected by reviewing the designated International Classification of Diseases-10 (ICD-10) diagnostic codes and medical records using relevant medications. Liver cirrhosis, or heart failure requiring admission,was also documented by reviewing the admission records of the patients. Presence of baseline chronic kidney disease was defined by a baseline eGFR less than 60 ml/min/1.73 m^2^, which isa common definition for stage 3 CKD [24]. Preoperative severe anemia was defined by a hemoglobin level less than 8 g/dL at baseline. The last laboratory values within 1 month before operation were regarded as the baseline levels.

Considering that the association between medication use and hyponatremia was well-reviewed [20], we collected information on the following medication use: ACE inhibitor or ARB, diuretics, NSAIDs, proton pump inhibitor, antiepileptic medication, thyroidal hormone replacement, antithyroidal medications (prophylthiouracil and methimazol), oral steroid, and serononin-norepinephrine reuptake inhibitor/selective serotonin reuptake inhibitor.

The following perioperative and intraoperative characteristics were reviewed: operation type, whether the surgery was an emergency operation, anesthesia type, risk stratification score including ASA classification [21] and NYHA classification [22] recorded by an anesthesiologist, total anesthesia time, total surgical time, type and amount of intraoperative administered fluids, intraoperative transfusion amount of RBC, and perioperative use of intravenous inotropic agents.

In addition, postoperative laboratory values and body weight changes within 7 days after the operation were collected. Postoperative AKI was staged by KDIGO clinical practice guideline for AKI [23], and also defined with sCr escalation within 7 days after operation.Information regarding postoperative intensive care unit admission and days of hospital stay wasalso documented.

### Outcome measurement

The primary renal outcome was defined as the event of progression to ESRD in the postoperative period. Progression to ESRD was defined by the start of maintenance dialysis or new progression to CKD stage 5 (eGFR < 15 ml/min/1.73 m^2^) [24]. The secondary outcome was a combination of additional long-term renal outcomes. The secondary outcome was defined as a composition of progression to ESRD, and sCr doubling/eGFR halving from baseline. All outcomes were defined and collected before the start of the analysis.

### Statistical analysis

Data are presented as frequencies and percentages for categorical variables and were analyzed by chi-squared tests. Continuous variables were expressed as the mean (standard deviation) or median scores (interquartile ranges) depending on the results of Shapiro-Wilk normality test. To assess risk factors for new-onset postoperative hyponatremia, we performed multivariable logistic regression with adjustment for variables that were regarded as clinically relevant and showed a significant difference between the study and the control groups. The renal outcomes were evaluated using the Cox regression hazard model. The association between postoperative sodium level and risk of the primary outcome was investigated by penalized spline analysis [25]. Multivariable analyses were adjusted per the following variables: age, sex, history of hypertension, diabetes mellitus, preoperative severe anemia, intraoperative use of normal saline, preoperative and postoperative serum creatinine level, main organ of surgery (bladder, prostate, ureter, or kidney), and surgery type (open, laparoscopic, endoscopic or robot-assisted). We used a complete case analysis method, as there were 253 cases of missing preoperative hemoglobin levels; no missing information was identified in other variables included in the multivariable analysis. All statistical analyses were performed using R package version 3.4.0 (R Development Core Team, Austria). Two-sided *p* values with a statistical significance level of 0.05 were used.

## Abbreviations

AKI: acute kidney injury
RBC: red blood cell
TUR-P: transurethral resection of prostate
eGFR: estimated glomerular filtration rate
CKD-EPI: Chronic Kidney Disease Epidemiology Collaboration
ACE I: angiotensin converting enzyme
ARB: angiotensin receptor blocker
NSAID: non-steroidal anti-inflammatory drugs
Na: sodium
OR: odds ratio
HR: hazard ratio
CI: confidence interval
sCr: serum creatinine
ESRD: end stage renal disease
